# Power Asymmetries and Punishment in a Prisoner’s Dilemma with Variable Cooperative Investment

**DOI:** 10.1371/journal.pone.0155773

**Published:** 2016-05-18

**Authors:** Jonathan E. Bone, Brian Wallace, Redouan Bshary, Nichola J. Raihani

**Affiliations:** 1 CoMPLEX, Physics Building, Gower Place, University College London, London, WC1E 6BT, United Kingdom; 2 Department of Economics, Drayton House, 30 Gordon Street, University College London, London, WC1H 0AX, United Kingdom; 3 Institut de Biologie, Eco-Ethologie, Université de Neuchâtel, Neuchâtel, CH-2000, Switzerland; 4 Department of Experimental Psychology, 26 Bedford Way, University College London, London, WC1H 0AP, United Kingdom; University of Exeter, UNITED KINGDOM

## Abstract

In many two-player games, players that invest in punishment finish with lower payoffs than those who abstain from punishing. These results question the effectiveness of punishment at promoting cooperation, especially when retaliation is possible. It has been suggested that these findings may stem from the unrealistic assumption that all players are equal in terms of power. However, a previous empirical study which incorporated power asymmetries into an iterated prisoner's dilemma (IPD) game failed to show that power asymmetries stabilize cooperation when punishment is possible. Instead, players cooperated in response to their partner cooperating, and punishment did not yield any additional increase in tendency to cooperate. Nevertheless, this previous study only allowed an all-or-nothing–rather than a variable–cooperation investment. It is possible that power asymmetries increase the effectiveness of punishment from strong players only when players are able to vary their investment in cooperation. We tested this hypothesis using a modified IPD game which allowed players to vary their investment in cooperation in response to being punished. As in the previous study, punishment from strong players did not increase cooperation under any circumstances. Thus, in two-player games with symmetric strategy sets, punishment does not appear to increase cooperation.

## Introduction

Punishment involves paying a cost in order to inflict harm on cheats or defectors [1]. Despite this cost, humans are often willing to invest in punishment in laboratory games involving two players (e.g. [2–5]) or multiple players (e.g. [6–10]). Subjective pleasure from punishing others seems to be one proximate mechanism underlying such actions [11, 12]. On a functional level, punishers may benefit from this investment if the target (or a bystander) behaves more cooperatively in future interactions [1,13,14]. Nevertheless, evidence that players do respond to punishment with cooperation is fairly mixed. Instead, previous studies have shown that players may often either continue to defect [3], or even retaliate [2, 3, 15, 16], when they are punished for cheating. It has been suggested that punishment is most likely to promote cooperation when it operates down a dominance hierarchy [1]; we test this hypothesis here.

Previous studies have typically assumed that all players are equal in terms of power, meaning that all players can punish for the same cost and impose the same fine on targets (e.g. [2, 4, 6, 7, 8, 10] but see [2, 17]). In reality, individuals are expected to vary in power, such that some players are able to inflict greater harm than their partners are able to reciprocate. When power asymmetries exist, it is expected that stronger players will punish weaker players but that weaker players will be unlikely to retaliate [1,13]. This prediction is borne out by data from the interspecific mutualism between cleaner fish (*Labroides dimidiatus*) and their reef-fish 'clients'. Cleaners provide a cleaning service to clients by removing skin ectoparasites [18]. Although cleaner fish obtain nutrients from eating these ectoparasites, they prefer to eat the client’s mucus, which constitutes 'cheating' [19]. If bitten, clients often terminate the interaction [20]. Cleaners sometimes work together in mixed sex pairs when cleaning a client. This creates a situation akin to a prisoner’s dilemma because whilst only one cleaner can reap the benefits of eating the client’s mucus, both share the cost of the interaction being terminated. A game theoretic analysis of this scenario demonstrated that for almost the entire parameter space, mutual defection is an evolutionary stable strategy [21]. Despite this, cleaner fish appear to have found a cooperative solution and pairs of interacting cleaner fish provide a better service to clients (more ectoparasite removal and less biting) than singletons [21]. The male fish are larger than the females and punish them by chasing them if they cheat—and punished females behave more cooperatively in the next interaction with that male [22]. However, females never punish or retaliate against cheating males, apparently due to the size difference [22–24], suggesting that power asymmetries might stabilize cooperation in these mixed-sex interactions.

Power asymmetries may also stabilize cooperation in human social dilemmas by making punishment from strong individuals more effective at promoting cooperation than in symmetric games. Nevertheless, recent empirical work which has incorporated power asymmetries into economic games has failed to detect any positive effect of power asymmetries on the effectiveness of punishment for strong players. One such study explored the effects of power asymmetries in a public goods game [17]. The authors found that asymmetries had no effect on punishment use, contributions to a public good or average payoffs. Although incurring punishment was shown to increase the contributions of low contributors, the authors did not test whether the effectiveness of punishment use was affected by power asymmetries. Moreover, in this study retaliation was not possible because players were not informed which of their peers punished them. A more recent study explored the effects of power asymmetries in a two-player iterated prisoner’s dilemma (IPD) game where retaliation was possible [2]. In this study, defecting players were more likely to cooperate in the next round if their partner cooperated, but punishment from the partner did not yield any additional benefits in either symmetric or asymmetric games. Moreover, counter to theoretical predictions, weak players were more likely to punish and retaliate in asymmetric games (i.e. against strong punishers) than in symmetric games. In fact, weak and strong players were equally likely to retaliate against strong partners [2].

One suggestion for why punishment from strong players failed to promote cooperation from weak partners in the previous study is because cooperation was a binary decision: players could only choose between cooperate or defect [2]. In such a setting, if the focal player's decision to cooperate (or defect) is conditioned on the partner's cooperative behavior in the previous round, then there is little scope for punishment to have an additional positive effect on the behavior of the target. In fact, cooperation in real-life situations often involves a variable rather than an all-or-nothing investment [25, 26]. For example, the cooperative allogrooming behavior exhibited in many animals (e.g. chacma baboons, *Papio cyanocephalus ursinus*) may last from just a few seconds, up for several minutes [27]. Similarly, in the cleaner fish example, although defecting is a binary outcome (bite client / do not bite client) cooperative investment is variable (duration of time 'cooperating' by removing ectoparasites; [21]). We therefore asked whether power asymmetries affected players’ average cooperation levels as well as their tendency to (i) increase their cooperation investment and (ii) retaliate in response to being punished when cooperation was a variable rather than binary investment.

In order to address this question we used a modified version of the IPD game, with variable rather than binary investments. The game was structured such that increasing investments yielded mutual benefits but each player could gain a larger benefit than the partner by choosing a slightly lower investment. Thus, the payoffs yielded the same incentive to defect as in the traditional prisoner's dilemma game. Asymmetries were incorporated into the game by allowing strong players to interact with weak players. As in previous work [2, 17] investing in punishment cost all players the same amount but strong players could inflict greater damage through punishing than weak players. In this study we used a two-player game as this allowed us to study the interaction between weak and strong players without the confounding effect of multiple other players’ behavior.

We predicted that players would be most likely to punish if their partner chose a lower cooperative investment than themselves (i.e. players would be more likely to punish defecting partners). Since previous work has found that in asymmetric games strong players were more likely to punish than weak players [2, 17], we expected to replicate this pattern in this study. Based on theoretical and empirical insights [1, 22, 23], we predicted that being punished by a strong partner would induce weak players to increase their investment in cooperation in the next round, though we did not expect to find the same effect of punishment when strong players were punished by either weak or by strong partners. Consequently, we envisaged that punishment from strong players would be more effective at promoting cooperation in asymmetric games than in symmetric games.

## Materials and Methods

### Experimental protocol

This research was approved by the University College London ethics board (project number 3720/001). All subjects remained anonymous so informed consent about the use of personal data was deemed unnecessary and was therefore waived by the University College London ethics board. The experiment took place over six sessions (one in May 2012, one in Nov 2012 and four in October 2014) in the experimental laboratory in the Department of Economics, University College London. The lab consists of twenty computers, which are visually partitioned. A total of 120 participants (71 women, 49 men, mean age ± se = 20.89 ± 0.20 years) were recruited from the student population to play a modified IPD game with a punishment option. Players interacted anonymously in pair-wise encounters by means of computer screens using the z-Tree [28] software. Each player played two games: one game with a partner of the same type as themselves (symmetric) and one game with a partner of a different type (asymmetric). The order in which players played symmetric and asymmetric games was counter-balanced. All players were paid a £5.00 show-up fee and their final score was summed over both games and multiplied by £0.02 to determine additional earned income. Thus, one game unit corresponded to £0.02. To allow for negative incomes while maintaining the £5.00 show-up fee, all players began each game with 100 units (£2.00) to play with. The average payment per player was £19.34 and the average session length was 90 minutes. Prior to the experiment, each player was given written instructions about the game structure and required to answer ten comprehension questions to verify their understanding of the game (S1 Appendix). The average score from the comprehension questions was 88%. Players were informed of the correct answers after the test.

The modified IPD game lasted 50 rounds. To avoid end effects [29], players were told that each game would last between 20 and 100 rounds. Players’ behaviour did not change abruptly towards the end of the game (S1 Fig), indicating that end effects were absent. Each round was split into two steps as follows:

Step 1: Both players simultaneously chose for how long they would like to cooperate with their partner. They could choose a time between zero and five seconds. For every second that both players cooperated they both got one unit. Whoever chose the shortest amount of time to cooperate for determined the duration of the interaction in that round and received a termination bonus of six units. If both players chose to interact for the same amount of time the interaction bonus was split into three units each. Hereafter, the amount of time a player chose to cooperate for will be called the players ‘cooperation level’. After both players made their choice, they were shown the cooperation level they chose, whether their partner chose a higher, lower or equal cooperation level (but not the exact cooperation level chosen by their partner) and each player's payoffs from this step. Payoffs for each decision in step 1 of the game are shown in Table 1.

**Table 1:** Payoffs accrued by player and partner for each decision combination in step 1.

		Partner cooperation level					
		0	1	2	3	4	5
Focal player cooperation level	0	(3, 3)	(6, 0)	(6, 0)	(6, 0)	(6, 0)	(6, 0)
	1	(0, 6)	(4, 4)	(7, 1)	(7, 1)	(7, 1)	(7, 1)
	2	(0, 6)	(1, 7)	(5, 5)	(8, 2)	(8, 2)	(8, 2)
	3	(0, 6)	(1, 7)	(2, 8)	(6, 6)	(9, 3)	(9, 3)
	4	(0, 6)	(1, 7)	(2, 8)	(3, 9)	(7, 7)	(10, 4)
	5	(0, 6)	(1, 7)	(2, 8)	(3, 9)	(4, 10)	(8, 8)

Payoff matrix for players (focal player, partner) in step 1 of each round of the experiment. The focal player's cooperation level is given in the rows and their partner's cooperation level is given in the columns.

Step 2: Players were then given the option of whether or not to punish their partner (described below). At the end of step 2, players were shown their own and their partner's choice and payoff from step 2, as well as the cumulative payoffs for both players for that round and their own total payoff (summed over all rounds). At the end of the first game, players were presented with the final scores and then randomly re-matched for the second game.

Players were randomly split into two types: weak and strong. Weak players punished with a 1: 1 fee to fine ratio, meaning that if they chose to punish their partner it would cost them one unit and it would also cost their partner one unit. Strong players punished with a 1: 6 fee to fine ratio, meaning that punishing their partner would cost them one unit but it would cost their partner six units. A 1: 6 fee to fine ratio was chosen because the termination bonus was six units; thus, if players who chose to cooperate for a smaller amount of time than a strong partner were punished their payoff was lower than if they had chosen an equal or higher cooperation level than their partner.

To rule out the possibility that less powerful players were being coerced into a position where they would do better if they could avoid interacting with the aggressor altogether (e.g. [30]), players could choose to not participate (opt-out) in any round of the game (e.g. as in [2, 31])). This option was presented in step one of each round of the game and meant that the current round was skipped and the next round then began as normal.

In order to avoid framing effects, neutral language was used. Player types “weak” and “strong” were replaced with “type 1” and “type 2”, “cooperate” was replaced with “interact” and “punish” and “don’t punish” were replaced by “option C” and “option D”. After both games had finished, all subjects were required to fill in a questionnaire to provide demographic information (S2 Appendix).

### Analyses

As in [2, 4], we decided to focus on the behavioral, rather than payoff, consequences of punishment. This is because, in the laboratory setting, individual payoffs are determined by the (largely) arbitrary costs and benefits associated with the options players are given in the game, as well as the number of rounds that players interact with one another. In a real world setting, any fitness benefits of punishment must stem from its ability to promote cooperative behaviour from partners or bystanders. Thus, in order to understand the contexts in which punishment is likely to be adaptive, it is more important to quantify the effect of punishment on targets' behaviour, rather than the total payoffs accumulated by the punisher. We used a series of generalized linear mixed models (GLMMs), to ask the following questions:

### 1. Did the mean cooperation level chosen by weak and strong players depend on whether they were in a symmetric or asymmetric game?

For each subject, we first calculated the mean cooperation level chosen in each game they played. The mean cooperation levels were then set as the dependent term in a GLMM with the following explanatory terms: ‘focal player type’ (a 2-level factor with levels weak/strong), ‘game type’ (a 2-level factor with levels symmetric/asymmetric) and the two-way interaction ‘focal player type x game type’. Sample size for analysis = 240 (120 players; each played 2 games). Rounds where either the focal player or their partner opted out were excluded from the analysis.

### 2. Did the propensity of weak and strong players to punish their partner depend on whether they were in a symmetric or asymmetric game?

Punishment was coded as a binary response term (player did not punish = 0; player punished = 1) and set as the dependent variable in a GLMM, with the following explanatory terms: ‘focal player type’ (a 2-level factor with levels weak/strong), ‘game type’ (a 2-level factor with levels symmetric/asymmetric) and the two-way interaction ‘focal player type x game type’. Instances of hypocritical or antisocial punishment (where the focal player punished the partner despite having chosen an equal or lower cooperation level than their partner, respectively) were not included in this model, leaving an N of 1735 rounds available for analysis.

### 3. Did being punished affect the likelihood that a player would increase their cooperation level in the next round in symmetric and asymmetric games?

Following [3], we compared the likelihood that focal players increased their cooperation level in round *n+1* after having been punished (or not) in round *n*. Whether or not focal players increased their cooperation level in round *n+1* was coded as a binary response term (player didn’t change or decreased cooperation level = 0; player increased cooperation level = 1) and set as the dependent variable in a GLMM, with the following explanatory terms: ‘focal player type’, ‘game type’, ‘partner punished in round *n’* (a 2-level factor with levels no/yes), all two-way interactions and the three-way interaction. If the focal player or their partner opted out of either round n or round n+1 then both round n and round n+1 were excluded from the analysis. In addition, instances of antisocial or hypocritical punishment were not included in this model. Thus, data were restricted to instances where the focal player chose a lower cooperation level than their partner in round *n*, leaving an N of 1655 rounds available for analysis.

### 4. Did being punished increase the likelihood that a player would retaliate in the next round in symmetric and asymmetric games?

We classed a player as retaliating if they punished a partner who chose a higher cooperation level than themselves in round *n + 1* (i.e. 'antisocially' punished the partner), having been punished (restricted to justified punishment) by that partner in round *n*. Whether or not focal players punished their (cooperative) partner in round *n+1* was coded as a binary response term (player did not punish = 0; player punished = 1) and set as the dependent variable in a GLMM, with the following explanatory terms: ‘focal player type’ (a 2-level factor with levels weak/strong), ‘game type’ (a 2-level factor with levels symmetric/asymmetric), ‘partner punished in round *n’* (a 2-level factor with levels no/yes), all two-way interactions and the three-way interaction. A positive effect of the term ‘partner punished in round *n’* would indicate that players retaliated in response to being punished. If the focal player or their partner opted out of either round n or round n+1 then both round n and round n+1 were excluded from the analysis. Data were restricted to instances where the focal player chose a lower cooperation level than their partner in round *n* and round *n+1*, leaving an N of 702 rounds available for analysis.

We also analysed the factors influencing players' decisions to opt out of rounds of the game. The results of this analysis are presented in S3 Appendix.

### Statistical methods

Data were analysed using R version 2.15.2 [32]. Generalized linear mixed models (GLMMs) with Gaussian error structure and identity link function were used for analysis 1 and GLMMs with binomial error structure and logit link function were used for analyses 2–4. GLMMs allow repeated measures to be fitted as random terms, thus controlling for their effects on the distribution of the data. For all models, player identity was included as a random term.

To determine the importance of explanatory terms in our models, we used an information theoretic approach with model averaging [33]. For each analysis we initially generated a global model. Following the specification of the global model, explanatory input variables were centered by subtracting their mean [34]. Centering of input variables allows averaging over models that include different interaction terms [34]. Sub-models were derived from the global model using the package MuMIn [35]. The degree of support for each sub-model was calculated using Akaike's Information Criterion corrected for small sample sizes (AICc [36]). The subset of top models was identified by taking the best model (the model with the lowest AICc value) and any models within 2AICc units of the best model. Each model in the top set was given Akaike weights, representing the probability that the given model is the true model (compared to other candidate models in the set) [37]. We computed the average parameter estimate (‘effect size’) and relative importance of each term from the top model set. The importance of a term can be thought of as the probability that the given term is a component of the best model; it is calculated by summing the Akaike weights of all models that include the term in question [37]. We only present the effect sizes from the top models. See S4 Appendix for R code and S5 Appendix for data.

## Results

### 1. Did weak and strong players, respectively, choose different mean cooperation levels in asymmetric and symmetric games?

**Fig 1:**
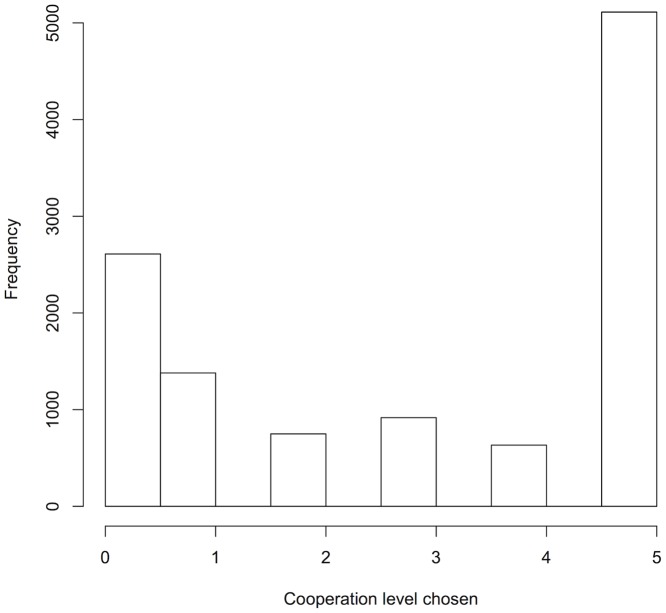
Histogram of cooperation levels chosen by players. Data exclude rounds where either player opted out.

**Fig 2:**
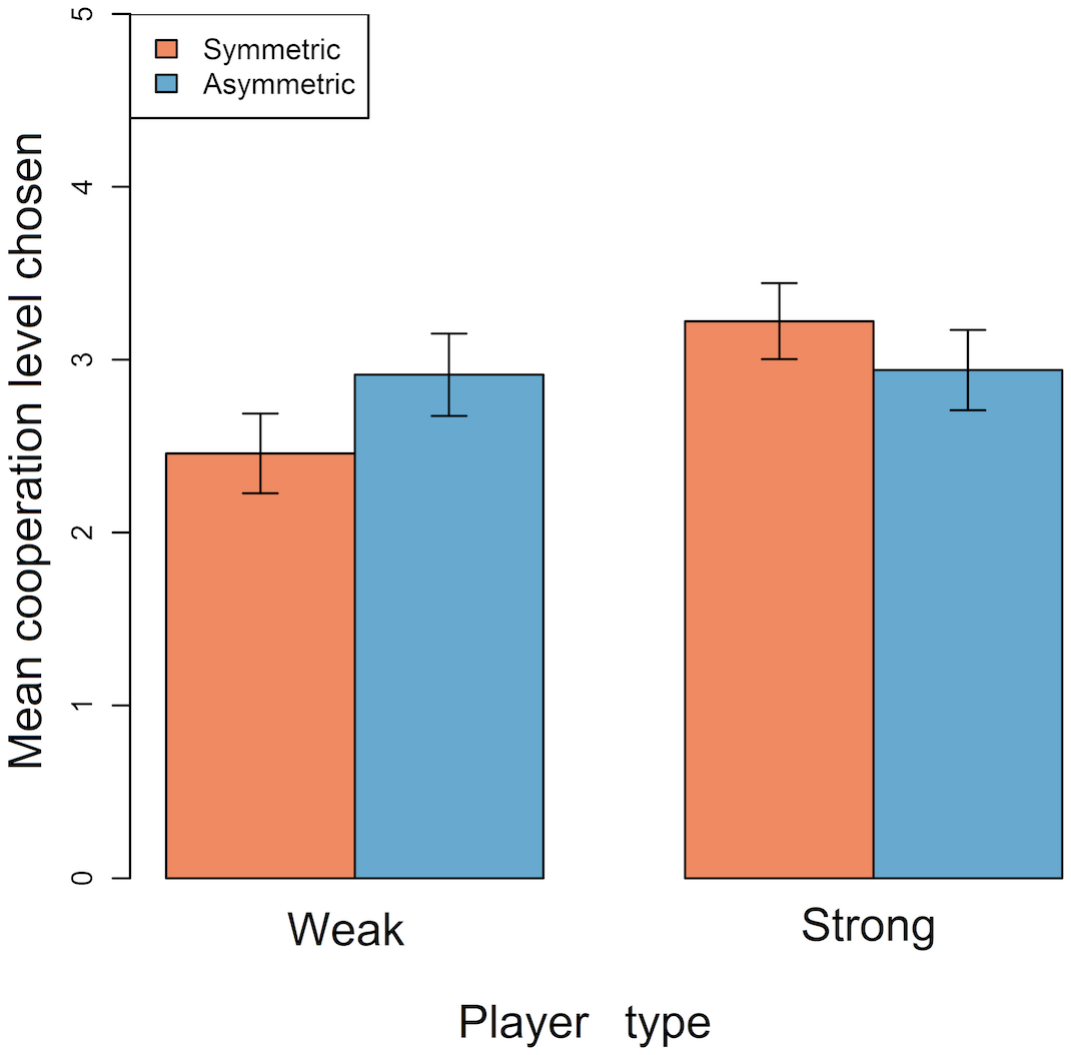
Barplot showing the mean (+/- SEM) cooperation levels chosen by weak and strong players in symmetric and asymmetric games. Data exclude rounds where either player opted out. Plots are generated from raw data.

Players often chose a non-zero cooperation level (mean proportion of rounds players chose non-zero cooperation level ± SE = 0.74 ± 0.03; Fig 1). Weak players chose higher cooperation levels in asymmetric games than in symmetric games (Fig 2; Tables 2 and 3). In contrast, strong players chose higher cooperation levels in symmetric games than in asymmetric games (Fig 2; Tables 2 and 3). Thus, both weak and strong players were most likely to cooperate if their partner was strong. In asymmetric games, weak and strong players chose equally high cooperation levels (Fig 2; Tables 2 and 3). In all conditions, mean cooperation levels increased slightly over the course of the game (S1 Fig). Cooperation levels appeared to increase more rapidly for strong players in symmetric games than for other players (S1 Fig).

**Table 2:** Summary data for weak and strong players.

	Weak players		Strong players	
	Symmetric	Asymmetric	Symmetric	Asymmetric
Cooperation level	2.51 (0.74–3.84)	3.32 (1.18–4.84)	3.85 (1.93–4.82)	3.24 (1.06–2.86)
Justified punishment	0.19 ± 0.04	0.14 ± 0.04	0.30 ± 0.05	0.41 ± 0.05
Hypocritical punishment	0.01 ± 0.00	0.02 ± 0.00	0.05 ± 0.00	0.06 ± 0.01
Antisocial punishment	0.03 ± 0.01	0.07 ± 0.03	0.16 ± 0.04	0.17 ± 0.04
Opted out	0.01 ± 0.00	0.07 ± 0.02	0.02 ± 0.01	0.01 ± 0.00

Summary data for cooperation level (median and IQ range) and mean proportion of instances where the focal player punished (justified / hypocritical / antisocial) and opted out (all means +/- SEM).

**Table 3:** Mean cooperation levels.

Parameter	Effect size	SE	Confidence Intervals	Importance
Intercept	2.88	0.14	(2.61, 3.15)	
Game type (asymmetric)	0.09	0.17	(-0.25, 0.42)	0.41
Focal player type (strong)	0.40	0.27	(-0.15, 0.94)	0.71
Game type x Focal player type	-0.74	0.34	(-1.41, -0.07)	0.41

Effect sizes, unconditional standard errors, confidence intervals and relative importance for parameters included in the top models investigating which factors affected the mean cooperation level chosen by the focal player in each game. Data from rounds where either the focal player or their partner opted out were excluded from the analysis.

### 2. Did the propensity of weak and strong players to punish their partner depend on whether they were in a symmetric or asymmetric game?

**Fig 3:**
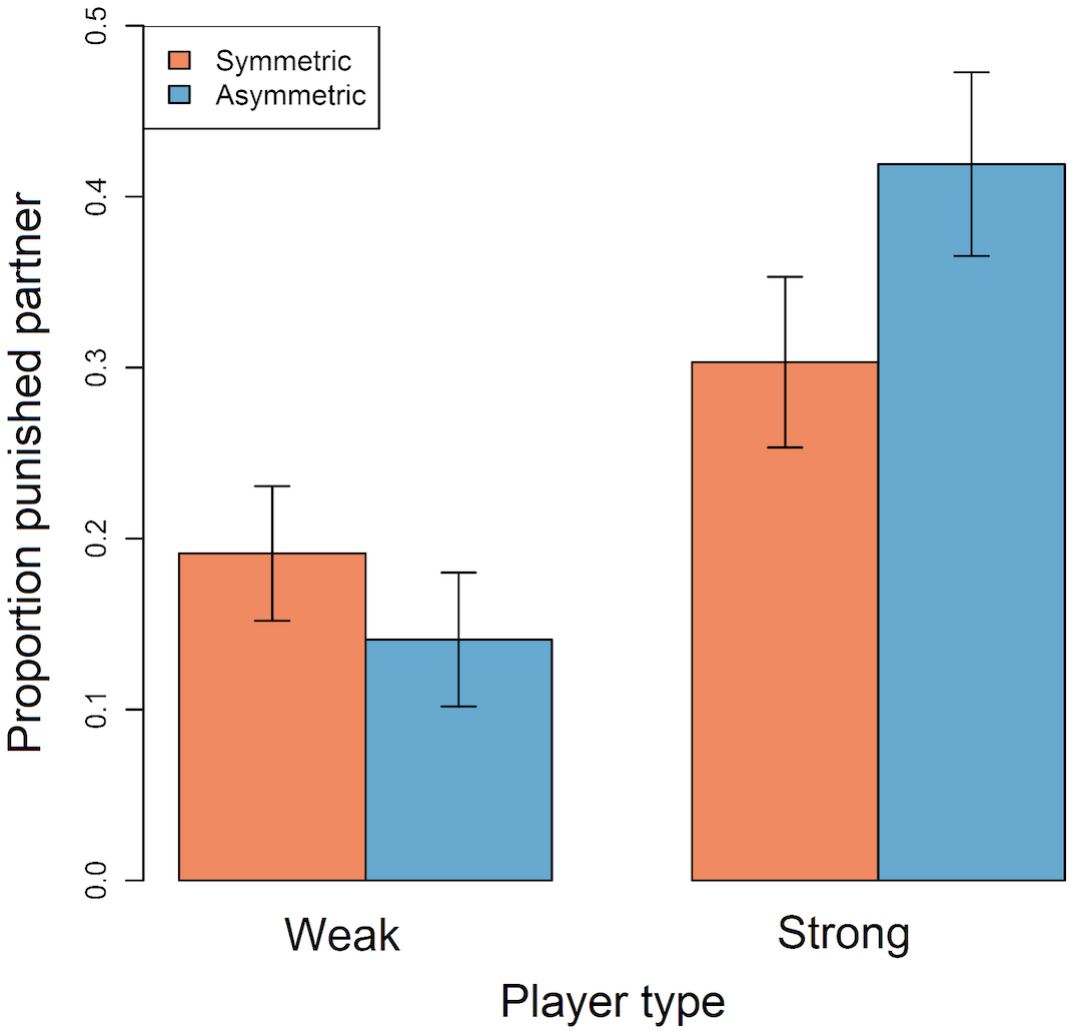
Barplot showing the mean (+/- SEM) proportion of instances in which weak and strong players punished their partner in symmetric and asymmetric games. Data were restricted to instances where the focal player chose a higher cooperation level than their partner and exclude rounds where either player opted out. Plots are generated from raw data.

In general, players were most likely to punish if their partner chose a lower cooperation level than themselves (‘justified punishment’) than if their partner chose an equal cooperation level (‘hypocritical punishment’) or a higher cooperation level (‘antisocial punishment’; Table 2). We investigated how the player's type (weak or strong) and game type (symmetric or asymmetric) affected their tendency to invest in justified punishment. Weak players were generally less likely to punish than strong players and, as expected, were more punitive in symmetric games than in asymmetric games (Tables 2 and 4; Fig 3). Strong players, on the other hand, were more likely to punish in asymmetric than symmetric games (Tables 2 and 4; Fig 3). Over the course of the game, the use of justified punishment decreased for all but strong players in symmetric games; for these players justified punishment actually increased throughout the game (S1 Fig). In all conditions, use of hypocritical and antisocial punishment started low but decreased to even lower levels over the game (S1 Fig).

**Table 4:** Factors affecting whether players punished their partners.

Parameter	Effect size	SE	Confidence Interval	Importance
Intercept	-1.84	0.22	(-2.31, -1.42)	
Game type (asymmetric)	0.09	0.19	(-0.28, 0.45)	1.00
Focal player type (strong)	1.70	0.43	(0.86, 2.59)	1.00
Game type x Focal player type	1.68	0.37	(0.96, 2.42)	1.00

Effect sizes, unconditional standard errors, confidence intervals and relative importance for parameters included in the global model investigating which factors affected whether focal players punished their partners (player did not punish partner = 0; player punished partner = 1). Only one top model was produced for this analysis. Data were restricted to instances where the focal player chose a higher cooperation level than their partner in that round; and rounds where neither the player nor their partner had opted out in the current or previous round (n = 1735).

### 3. Did being punished affect the likelihood that a player would increase their cooperation level in the next round in symmetric and asymmetric games?

**Fig 4:**
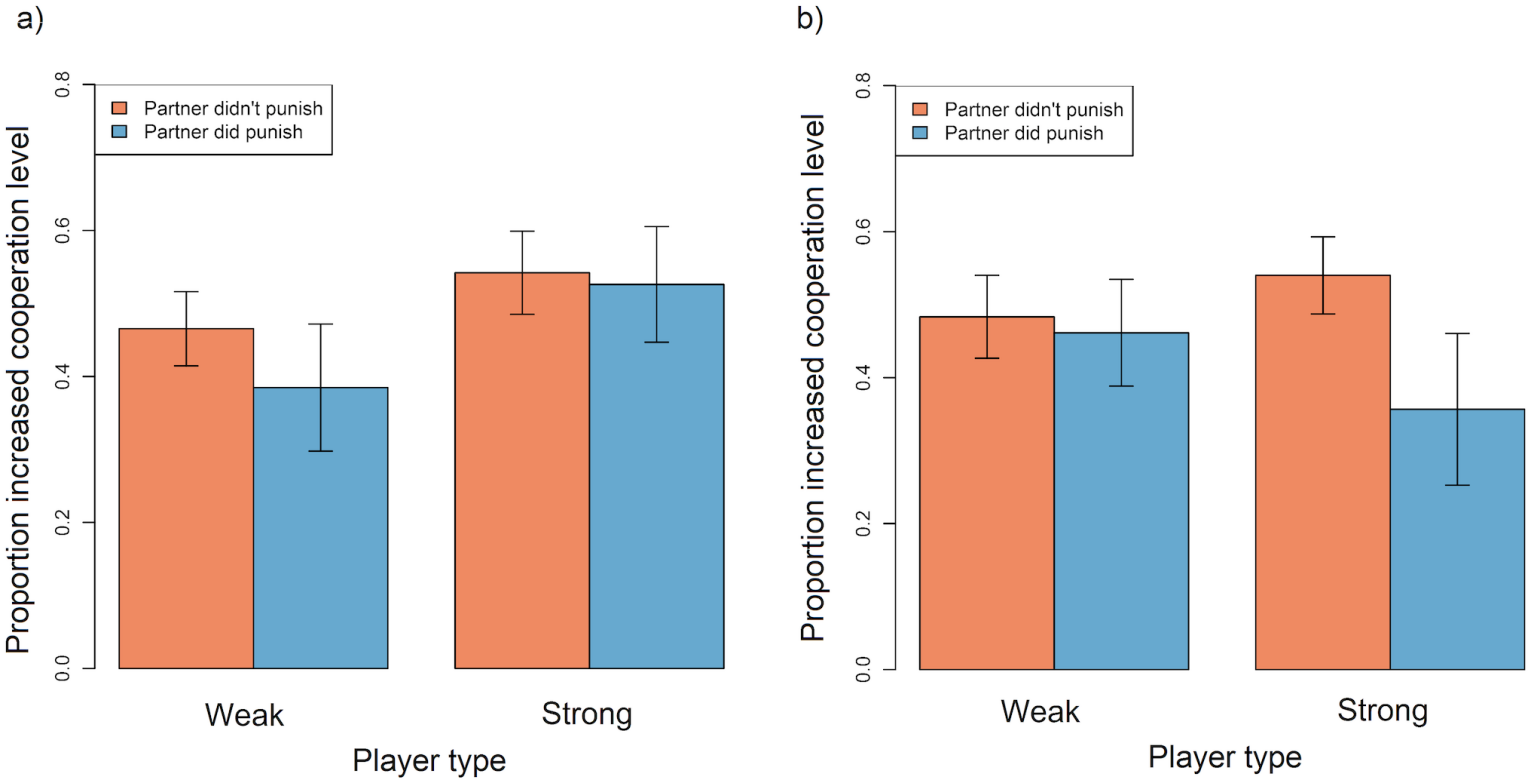
Barplot showing the mean proportion of instances in which weak and strong players in a) symmetric games and b) asymmetric games increased their cooperation level in round *n+1* relative to round *n*, according to whether or not they were punished by their partner in round *n*. Data were restricted to instances where the focal player chose a lower cooperation level than their partner in round *n* and exclude instances where either player opted out in round *n* or *n+1*. Error bars represent standard errors. Plots are generated from raw data.

Justified punishment had no discernible effect on the target's tendency to increase their cooperation level in the next round, regardless of whether they were weak or strong or the game type (Table 5; Fig 4).

**Table 5:** Factors affecting whether players cooperated increased cooperation level.

Parameter	Effect size	SE	Confidence Interval	Importance
Intercept	-0.09	0.14	(-0.37, 0.19)	
Partner punished in round *n* (yes)	-0.19	0.16	(-0.50, 0.12)	0.43
Focal player type (strong)	0.31	0.29	(-0.25, 0.88)	0.31
Game type (asymmetric)	0.10	0.13	(-0.17, 0.36)	0.22

Effect sizes, unconditional standard errors, confidence intervals and relative importance for parameters included in the top models investigating which factors affected whether or not focal players increased their cooperation level in round *n+1* relative to round *n* (player didn’t change or decreased cooperation level = 0; player increased cooperation level = 1). Data were restricted to instances where the focal player chose a lower cooperation level than their partner in round *n* and exclude instances where either the focal player or their partner opted out in round *n* or round *n+1 (*n = 1655).

### 4. Did being punished increase the likelihood that a player would retaliate in the next round in symmetric and asymmetric games?

**Fig 5:**
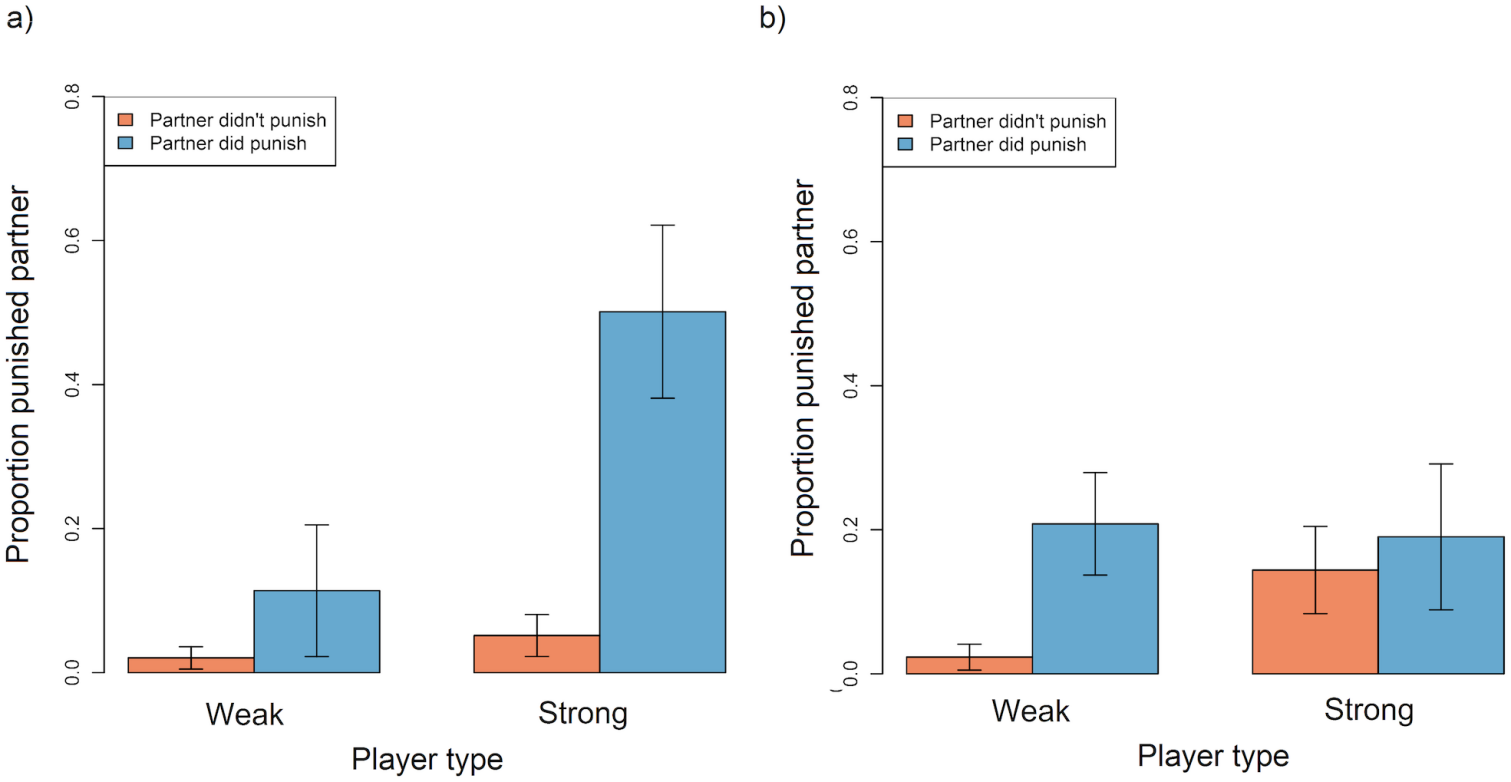
Barplot showing the mean proportion +/- SE of instances that weak and strong players in a) symmetric and b) asymmetric games punished a more cooperative partner in round *n+1*, according to whether they were punished by their partner in round *n*. Data were restricted to instances in which the focal player choose a lower cooperation level than their partner in round *n* and *n+1*. Rounds where either player opted out in round *n* or *n+1* were also excluded. Red bars represent antisocial punishment whereas blue bars can be interpreted as retaliation for punishment previously received. Plots are generated from raw data.

Weak and strong players both retaliated in response to justified punishment (i.e. they were more likely to punish a cooperative partner if this partner had punished them in the previous round than when the partner had not punished in the previous round; Table 6; Fig 5). Contrary to our predictions, neither the focal player’s type nor game type had an effect on whether players retaliated against punitive partners (Table 6; Fig 5). Although strong players in symmetric games appeared to retaliate more frequently than players in other conditions (Fig 5), the 3-way interaction between the focal players type, the game type and whether or not the focal player was punished by their partner in the previous round was not a component of the top models, meaning evidence for this effect is weak.

**Table 6:** Factors affecting whether players retaliated against punishers.

Parameter	Effect size	SE	Confidence Interval	Importance
Intercept	-3.83	0.50	(-4.81, -2.86)	
Partner punished in round *n* (yes)	2.71	0.46	(1.81, 3.61)	1.00
Focal player type (strong)	1.75	0.67	(0.43, 3.06)	1.00
Game type (asymmetric)	0.41	0.41	(-0.39, 1.21)	0.44
Game type x partner punished in round *n*	-1.13	0.84	(-2.77, 0.51)	0.21
Partner punished in round *n* x Focal player type	0.19	0.87	(-1.51, 1.89)	0.15

Effect sizes, unconditional standard errors, confidence intervals and relative importance for parameters included in the top models investigating which factors affected whether a focal player punished a cooperative partner (player did not punish = 0; player punished = 1). Data were restricted to instances the focal player chose a lower cooperation level than their partner in round *n* and round *n+1*. The term 'partner punished in round n' describes whether the punishment can be interpreted as retaliation (i.e. focal player retaliating against a punitive partner) or antisocial punishment (i.e. focal player punishing a cooperative partner).

## Discussion

It has been suggested that asymmetries in power may stabilize cooperation by making punishment operating down a dominance hierarchy more effective at promoting cooperation [1,13, 22, 23]. However, a previous empirical study failed to support the idea that power asymmetries allow punishment to promote cooperation in humans [2]. This failure was potentially due to decisions involving an all-or-nothing—rather than a variable—cooperation investment [2]. As such, here we used a modified IPD game to test how power asymmetries affect the use of punishment and its effectiveness at promoting cooperation when both variable cooperation investment and retaliation were possible. As expected, players were most likely to punish partners that chose a lower cooperation level than themselves and, as observed in previous studies [2, 38], strong players were more likely than weak players to punish their partners. Strong players also preferentially targeted weak partners for punishment. Weak players, on the other hand, were less likely to punish when faced with a strong partner. These findings support the prediction that punishment is most likely to operate down a dominance hierarchy [1].

Other findings did not support our predictions. For example, we predicted that weak players would respond to punishment from strong partners with increased cooperation in the next round, whereas we did not expect such an effect when strong players were punished by either weak or strong partners. However, we found that in all conditions, punishment had no effect on a target's tendency to increase their cooperation level in the next round. Moreover, players' tendency to retaliate against punishers did not vary with game type for either strong or weak players. These findings are consistent with previous work which incorporated power asymmetries into an IPD [2] and suggest that, in two-player prisoner's dilemma games with symmetric strategy sets (i.e. where defection is an option for both partners, [20]), punishment might not be an effective strategy for motivating partners to cooperate (see also [4, 39] for similar findings).

Instead, the fact that punishment rarely induced partners to cooperate and often provoked retaliation supports the idea that conditionally cooperative strategies might often outperform punitive strategies in two-player games [40], so long as both players have the option to defect in response to a defector (e.g. see [20]). If strategy sets are asymmetric, such that one class of player cannot defect when faced with a defector, then punishment might effectively promote cooperation in two-player games [20]. Conditionally cooperative strategies are expected to be less effective in multiplayer games where defection harms cooperative partners as well as defectors [13]. Punishment may therefore be more effective in multiplayer games than in two-player games (e.g. [41]).

Empirical studies have shown that players often increase their cooperation levels [2, 42] (or reduce investment in third-party punishment, [43]) if they know their peers are able to punish them. Importantly, the mere threat of punishment [44], even when it is not realised, seems to be sufficient to motivate this change in behaviour. The findings from the current study support the idea that punishment threats may be important in the context of cooperation. Weak players generally chose higher cooperation levels in asymmetric games than in symmetric games, which suggests that the threat of punishment from a strong partner may have deterred weak players from defecting more effectively than the threat of being punished by a weak partner. These findings are also consistent with [2] where it was found that while incurring punishment did not elicit cooperation from targets in the following round, players were generally more cooperative if their partner was strong. Although the implications of this finding were not discussed in the earlier study, together these studies suggest that the threat of punishment from a strong player may be sufficient to promote cooperation, even if actual punishment has no effect. Further work is clearly required to understand how punishment threats even when punishment is never implemented) affect social behaviour in humans and other species.

It is possible that strong players would take the threat of being punished by a strong partner less seriously because they possess a credible threat of retaliation of their own. If this were the case, then we would have expected that strong players would cooperate less than weak players when paired with a strong partner. However, strong players in symmetric games actually chose higher mean cooperation levels than weak players in asymmetric games, suggesting that the threat of being punished by a strong partner deterred cheating regardless of whether the focal player was weak or strong. Perhaps more puzzling is the finding that weak and strong players were equally cooperative in asymmetric games. It is possible that the high levels of cooperation exhibited by strong players in asymmetric games were a result of conditionally cooperative strategies [45], whereby strong players behaved cooperatively because they believed their weak partners would cooperate as well; rather than because they feared being punished.

Although weak players were less likely to punish strong partners than weak partners, this effect was relatively small, and weak players often punished even in asymmetric games. The fact that weak players punished and retaliated against strong partners in these human experiments but in the cleaner fish system, females never punish or retaliate against larger, dominant males [21–23], may be associated with the different costs associated with provoking aggressive responses from a more dominant partner. For example, in this experiment and [2], punishment (or retaliation) from a strong partner meant losing a known and relatively small amount of money. However, for female cleaner fish the cost of associated with provoking punishment (or retaliation) from a male fish is unknown and could potentially be fatal. In addition, for female fish, retaliating against a punitive male carries a risk of escalating aggression [24] which was not possible in our game because opportunities to punish and the impact of punishment were fixed. Indeed, one might argue that allowing players to make variable investments in cooperation but restricted them to making a fixed investment in punishment is unrealistic, since in real-world scenarios individuals can presumably choose how much to allocate to punishment (e.g. see [24]). One might instead expect to see individuals initially signal disapproval with a defector's behaviour by imposing a small punishment fine, but then escalate punishment in response to continued defection from a partner. Whether a flexible punishment technology, which gives players the options to both start small and escalate to much higher fines, might be more effective at promoting cooperation would be an obvious next step to explore in this regard.

The relatively small costs associated with being punished may also in part explain the ineffectiveness of punishment at promoting cooperation in both this study and [2]. Crucially, ‘cheating’ players received a higher payoff than their partner even if they were punished by a strong partner. Thus, if players are motivated by a desire to out-compete their partner as suggested in previous work (e.g. [46]), avoiding punishment may not have proved a sufficient incentive for players to behave more cooperatively. Future work should ask whether power asymmetries promote cooperation when the costs associated with retaliation are larger or when punishment impact can escalate.

An alternative explanation for why weak players readily punished and retaliated against strong partners is that although we incorporated power asymmetries into the game these may have failed to translate into dominant and subordinate social roles in players’ minds. This could stem from the use of neutral language in the game instructions given to participants. For example, although players were aware of the different payoff consequences of actions performed by the two player types, weak and strong players were referred to as ‘Type 1’ and ‘Type 2’ respectively (S1 Appendix). It is possible that these labels were not salient enough to elicit the behavioural responses we expected. This is a stark contrast to the famous Stanford prison experiment [47] where participants were randomly assigned the role of a prisoner or guard. In this experiment, effort was taken to make the situation as realistic as possible (e.g. guards were given sticks and uniforms and prisoners were arrested by the police department, deloused, forced to wear chains and prison garments). Under these conditions, within a short time both guards and prisoners settled into their new roles leading to extreme transformations of character [47]. Other studies have shown that using loaded language like ‘bribe’ and ‘punish’ rather than neutral equivalents can produce significant changes in subjects' behaviour in economic games (e.g.[48]). Although neutrally worded instructions have become a mainstream practice in behavioural experiments, it has been argued that it may be more useful to explore the effect of context rather than attempting the impossible goal of excluding it from experiments [49]. Future work could therefore explore how players behave in similar experiments when they are explicitly told that they are playing the role of a dominant or subordinate individual.

As observed in previous work [2], weak players in asymmetric games were considerably more likely to opt out than players in other conditions. In addition, weak players were more likely to opt out of rounds if they were previously punished by a strong partner than when they were not punished (S3 Appendix). This suggests that weak players sometimes avoided further punishment from strong players by withdrawing from the game rather than by increasing their cooperation level. In this study, players (especially weak players) opted out less often than in our previous study [2] (players opted out of around 3% of rounds in this study vs. 11% of rounds in [2]). We suggest that players opted out less frequently in this study than in the previous study because, in the current study, players could earn a positive payoff even if they chose a higher cooperation level than their partner (i.e. their partner defected). This was not the case in the previous study. Thus, unless they were the target of hypocritical or antisocial punishment, players in this study were always absolutely (if not relatively) better off participating rather than opting out.

To summarize, we found that in a variable investment IPD, power asymmetries did not make punishment from strong players more effective at promoting cooperation in comparison to symmetric games. In fact, punishment provoked retaliation, rather than cooperation in all conditions. This finding supports previous work which has suggested that in a two-player setting, conditional cooperation may sustain cooperation more effectively than punishment [40]. We suggest that future research could explore the effect of power asymmetries when aggression can escalate. In addition, we propose that further work is required to understand how the threat of being punished influences players' behavior even when punishment is never implemented.

## Supporting Information

S1 Appendix Experimental instructions given to participants. (DOC)

S2 Appendix Demographic data. (DOC)

S3 Appendix Analysis of opt-out decisions. (DOC)

S4 Appendix R code used for analyses. (DOC)

S5 Appendix Raw data used for analysis. (CSV)

S1 Fig Cooperation and punishment over successive rounds. (DOC)
