# Supplementary material for: Power Asymmetries and Punishment in a Prisoner’s Dilemma with Variable Cooperative Investment
Source: PLoS One. 2016 May 18;11(5):e0155773. doi: 10.1371/journal.pone.0155773 (PMC4871419; doi:10.1371/journal.pone.0155773)
Supplement: S1 Appendix — (DOC) [file pone.0155773.s001.doc]

**S1 Appendix. Experimental instructions**

Welcome and thank you for participating in this experiment.

Please be quiet during the entire experiment. **Do not talk to your neighbours and do not try to look at their screens.** If you have any questions, please raise your hand. We will come to you and answer it privately.

This experiment is about decision-making. **You will be randomly assigned the role as either a “type 1” or a “type 2” player.** You will keep this role throughout the experiment. You will play a two-player game twice. Each game will last a predetermined number of rounds **(between 20 and 100).**

In each game you will be **randomly matched with a different person in the room.** You will play with the same person for the duration of each game. Therefore, you will play with two people in total. In one game you will play with someone of the same type as yourself and in one game you will play someone of a different type to yourself. **You will remain anonymous throughout the experiment** and will be identified only by the name in the top left corner of your computer screen.

**Before playing the games you will answer some questions about the experiment.** The purpose of this is to make sure that everybody fully understands the rules of the experiment before we start so please make sure you read these instructions carefully.

Depending on your decisions and the decisions made by the other player in each of the two games you play, you will able to earn a considerable amount of money. The scores you receive in the game will be given in units **(1 unit= 2p).**

Everyone will receive **a show up fee of £5**. In addition, you will be given **an additional 100 units (£2.00) to play with at the start of each game**. Depending on the decisions made by you and the other player during each game you will gain or lose units from this initial amount. After the experiment has finished, your units will be converted to real money and you will be paid your earnings together with the show-up fee. In total this could be as much as £29.

**Payment**: Payment will happen after both games have been played.

**The Game:**

Each round will be split into two steps. **In each step you will be asked to make a decision. The decisions you make will affect the income that you and the other player receive for that round.** There is a time limit of **15 seconds** on each step (shown in the top right corner of the screen). It is important you make a choice within this time, if not a **default choice will be picked for** **you** and you will move on to the next step.

Each of these steps will now be explained to you in detail.

**Step 1:** You and the other player **simultaneously choose how long you would like to interact for** (between 0 and 5 seconds). To do this you will **select the button corresponding to the amount of time you decide to interact for** then click OK. Whoever chooses the smallest time determines the duration of the interaction in that round. For example, if you choose to interact for 3s and the other player chooses to interact for 5s the interaction will last for 3s. For **every second** (s) that you and the other player interact, both players will get an income of **+1 unit.**

If one player chooses to interact for less time than the other they will also receive **a termination bonus of +6 units.** If both players choose to interact for the same amount of time the termination bonus of +6 units is split between the two players so that each player receives **+3 units.**

**You will also have the option of “Do not participate in this round”.** If either player chooses “Do not participate in this round” both players will skip the second step and neither player will gain or lose any units in this entire round. The next round will begin as normal.

Some examples are given below:

If both you and the other player choose to interact for 3s you will both receive +6 units (+3 for interacting for 3s, +3 split termination bonus = +6 total).

If you choose to interact for 2s and the other player chooses to interact for 3s you will receive +8 units (+2 for interacting for 2s, +6 termination bonus = +8 total).

If you choose to interact for 5s and the other player chooses to interact for 0s you will receive 0 units 0 for interacting for 0s, 0 termination bonus = 0 total).

**Step 2:** ***If either player chose “Do not participate in this round” in step 1 then this step is skipped.***

The decisions and scores from step one are presented to both players. You must both then decide whether you would or would not like to reduce the other player's income at a cost to yourself by choosing between options “A” or “B”, respectively.

Option “A” = reduce the other player's income at a cost of 1 unit to yourself.

Option “B” = do nothing (neither you nor the other player will gain or lose any points).

**The number of units that the other player’s income is reduced by when you choose option “A” varies depending upon what “type” of player you were assigned as at the start of the experiment.**

Type 1 player: If you choose “A” then you will lose 1 unit, and the other player will lose 1 unit.

Type 2 player: if you choose “A” you will lose 1 unit, and the other player will lose 6 units.

After you have made a decision you and the other player are presented with each other’s choices and incomes from step 2 as well as your total income for the round. You will also be told your total score for the current game.

Your overall income in step 2 is determined by the addition of the income from both your decision and the other player's decision.

Your income for the round will be determined by the addition of your incomes from step 1 and step 2.

**The total number of units that you have at the end of these games will determine how much money you have earned.** Therefore, the additional money you and the other player each earn depends on the options you choose in each step of the game. **However, the final scores of the other players do not matter for your final earnings.**

If your total score drops to 0 units, you will not be able to play for the remaining rounds of the game of the current game.

At the end of both games, your total earnings will be computed. If you finish with a total score of 0 units over the two games, you will walk away with just the £5 show up fee. If you have more than 0 units, you will earn extra money at the exchange rate of 1 unit= 2p. The maximum extra amount that you can earn will be £24.

We will distribute a questionnaire at the end of the experiment that will ask some basic information about you.

Please take your time to read through the instructions again and if you have any questions raise your hand. In a few minutes we will begin the questionnaire followed by the games.

**Comprehension questions**

All participants were required to answer the following comprehension questions before playing the game. If they answered a question incorrectly they were shown the correct answer. The possible answers are shown in parentheses.

1. Each game will last a predetermined number of rounds, between what? (10-200 / 20-100 / 0-50)

2. How many games will you play in this experiment? (1 / 5 / 2)

3. If you finish the experiment with a total score of 500 units, how much money will you earn ontopofthe£5showupfee(1unit=2p)?(£10 /£5/£80)

4. If your total score drops to 0 or below in a game what will happen? (You will lose your show up fee / You will have to leave the room / You will not be able to play the remaining rounds of the current game)

5. If both you and the other player chose to interact for 3 seconds, how many units will you get in step 1? (1 / 3 / 6)

6. If you chose to interact for 4 seconds and the other person chose to interact for 5 seconds, how many units will you get in step 1? (10 / 1 / -6)

7. If you choose to interact for 2 seconds and the other player chooses to interact for 1 second, how many units will you get in step 1? (2 / 7 / 1)

8. If you are a "type 1" player and choose option "A" in step 2, how many units will be deducted from the other players income? (1 / 3 / 5)

9. If both you and the other player are "type 2" players and you both choose option "A" how manyunitswillyougetinstep2? (1/-7/2)

10. If both you and the other player are "type 1" players, you both choose to interact for 5 seconds in step 1 and both choose option "B" in step 2, what will your income be in that round? (8/2/-5)
