# Supplementary material for: Power Asymmetries and Punishment in a Prisoner’s Dilemma with Variable Cooperative Investment
Source: PLoS One. 2016 May 18;11(5):e0155773. doi: 10.1371/journal.pone.0155773 (PMC4871419; doi:10.1371/journal.pone.0155773)
Supplement: S2 Appendix — (DOC) [file pone.0155773.s002.doc]

**S2 Appendix. Demographic data**

After the game had finished, all participants were required to answer the following demographic questions.

1. What is your gender?

2. How old are you?

3. What is your country of origin?

4. What is your subject of study?

**S1 Table.**

| Parameter | Weak | Strong |
| --- | --- | --- |
| Age | Mean (SE) = 20.7 (0.3) | Mean = 21.1 (0.3) |
|  | Median (IQR) = 20 (19-22) | Median (IQR) = 21 (20-22) |
|  |  |  |
| Gender (F, M) | 27, 33 | 38, 22 |
|  |  |  |
| Country of origin | Bangladesh = 1 | Australia=1 |
|  | Belarus = 1 | Azerbaijan=1 |
|  | Canada = 1 | China=9 |
|  | China = 4 | Czech Republic = 1 |
|  | Czech Republic = 1 | France = 1 |
|  | Denmark = 1 | Germany=1 |
|  | France = 1 | Greece=2 |
|  | Greece = 1 | Hong Kong=5 |
|  | Hungary = 2 | Iceland=1 |
|  | India = 4 | India=1 |
|  | Indonesia =1 | Indonesia =1 |
|  | Japan=1 | Israel=1 |
|  | Jordan=1 | Italy=1 |
|  | Malaysia=6 | Malaysia=9 |
|  | Nepal=1 | Poland=1 |
|  | Pakistan=2 | Romania=4 |
|  | Peru=1 | Russia=1 |
|  | Poland=2 | Saudi Arabia=1 |
|  | Russia=2 | Singapore=4 |
|  | Singapore=5 | Spain=1 |
|  | Switzerland=1 | Taiwan=2 |
|  | UK=14 | Thailand=1 |
|  | USA=4 | UK=8 |
|  | Zimbabwe=1 | USA=1 |
|  |  | Vietnam=1 |
|  |  |  |
| Subject studied | Anthropology=1 | Anthropology=1 |
|  | Biochemistry=2 | Archaeology=1 |
|  | Biology=2 | Architecture=1 |
|  | Biomedical Sciences=2 | Art & Sciences=3 |
|  | Chemical Engineering=2 | Astrophysics=1 |
|  | Chemistry=1 | Biology=1 |
|  | Economics=5 | Brain & Mind=1 |
|  | Economics & Business=1 | Chemical Engineering=1 |
|  | Electronic Engineering=2 | Civil Engineering=2 |
|  | Engineering=4 | Computer Science=1 |
|  | English=1 | Digital humanities=1 |
|  | Fine Art=1 | Economics=7 |
|  | French & German =1 | Engineering=2 |
|  | Geography=2 | Fine Art=1 |
|  | History=2 | Geography=1 |
|  | History of Art=1 | History=1 |
|  | History Philosophy Science=1 | Human Genetics=1 |
|  | Human Genetics=1 | Human Sciences=2 |
|  | Human Sciences=1 | Infrastructure Investment =1 |
|  | Language& Culture=1 | Language& Culture=3 |
|  | Translation theory=1 | Law=5 |
|  | Management=1 | Materials Science =1 |
|  | Mechanical Engineering=1 | Mathematics =2 |
|  | Modern Languages=1 | Medicine =3 |
|  | Natural Sciences =1 | Modern Languages=1 |
|  | Pharmacogenetics=1 | Pharmacology=1 |
|  | Pharmacy=2 | Pharmacy=4 |
|  | Philosophy=1 | Philosophy=1 |
|  | Philosophy & Economics=1 | Physics=2 |
|  | Physics=4 | Psychology=3 |
|  | Political Science =4 | Speech Science =1 |
|  | Psychology=1 | Statistics =2 |
|  | Systems Engineering=1 | Statistics & Economics = 1 |
|  | Urban Planning =1 | Urban Planning =1 |
|  |  | Zoology=1 |

**S1 Table.** Information on mean, median values and sample sizes for participant demographic data.
