# Supplementary material for: Power Asymmetries and Punishment in a Prisoner’s Dilemma with Variable Cooperative Investment
Source: PLoS One. 2016 May 18;11(5):e0155773. doi: 10.1371/journal.pone.0155773 (PMC4871419; doi:10.1371/journal.pone.0155773)
Supplement: S3 Appendix — (DOC) [file pone.0155773.s003.doc]

**S3 Appendix. Analysis of opt out decisions**

Initially we asked whether the propensity of weak and strong players to opt out of rounds depended on whether they were in a symmetric or asymmetric game. Generalised linear mixed models (GLMMs) with model averaging were used for this analysis (as described in the main text). The response term encoded whether the focal player opted out of the round round (player didn’t opt out = 0, player opted out = 1). The explanatory variables included in this model were: ‘focal player type*’* (a 2-level factor with levels weak/strong), ‘game type’ (a 2-level factor with levels symmetric/asymmetric) and the two-way interaction ‘player type x game type. All data were included in this analysis (N = 12000 rounds). This analysis showed that although opting out of rounds was generally rare, weak players in asymmetric games were more likely to opt out than players in other conditions (Table 2; S2 Table).

We then asked whether players responded to being punished in round *n* by opting out of round *n+1*. Data were restricted to instances where the focal player had chosen a lower or cooperation level than their partner in round *n* (i.e. the effect of antisocial or hypocritical punishment on target focal player opting out was not measured). Due to the small proportion of rounds in which strong players or weak players in symmetric games opted out (Table 2), we did not have the statistical power to test what factors affected opt out decisions for these players. Thus, data were restricted to weak players in asymmetric games (448 rounds were included in this analysis). Whether or not the focal player opted out of round *n+1* was coded as a binary response term (player didn’t opt out = 0, player opted out = 1) and set as the dependent variable in a GLMM. The only explanatory variable included in this model was: ‘partner punished in round *n’* (a 2-level factor with levels no/yes). This analysis showed that weak players in asymmetric games were more likely to opt out if they were punished by their partner in the previous round (S3 Table).

| Parameter | Effect size | SE | Confidence Interval | Importance |
| --- | --- | --- | --- | --- |
| Intercept | -9.88 | 1.01 | (-11.91, -7.94) |  |
| Focal players type (strong) | 0.18 | 0.93 | (-1.66, 2.05) | 1.00 |
| Game type (asymmetric) | 1.17 | 0.19 | (0.79, 1.56) | 1.00 |
| Focal players type x Game type | -4.42 | 0.39 | (-5.21, -3.69) | 1.00 |

**Table S2.** Effect sizes, unconditional standard errors, confidence intervals and relative importance for parameters included in the top models investigating which factors affected whether the focal player opted out of rounds of the game (player did not opt out = 0; player opted out = 1).

| Parameter | Effect size | SE | Confidence Interval | Importance |
| --- | --- | --- | --- | --- |
| Intercept | -10.18 | 2.85 | (-17.08, -5.86) |  |
| Partner punished in round *n* | 3.32 | 1.52 | (0.93, 7.21) | 1.00 |

**Table S3.** Effect sizes, unconditional standard errors, confidence intervals and relative importance for parameters included in the top models investigating which factors affected whether the focal player opted out of rounds of the game (player didn’t opt out of round n+1 = 0, player opted out of round n+1 = 1). Data are restricted to weak players in asymmetric games in instances where the focal player chose a lower cooperation level than their partner in round n.
