# Supplementary material for: Power Asymmetries and Punishment in a Prisoner’s Dilemma with Variable Cooperative Investment
Source: PLoS One. 2016 May 18;11(5):e0155773. doi: 10.1371/journal.pone.0155773 (PMC4871419; doi:10.1371/journal.pone.0155773)
Supplement: S4 Appendix — (DOC) [file pone.0155773.s004.doc]

**S3 Appendix**. R code used to fit generalised linear mixed models

library(MuMIn)

library(arm)

library(lme4)

read.csv(file="Data.csv",head=TRUE,sep=",")->data

data[data$Player.OptOut==0,]->data

data[data$Partner.OptOut==0,]->data

#Model 1

data[data$Players.type==0&data$Game.type==0,]->dataweaksym data[data$Players.type==1&data$Game.type==0,]->datastrongsym data[data$Players.type==0&data$Game.type==1,]->dataweakasym data[data$Players.type==1&data$Game.type==1,]->datastrongasym

rowSums(table(dataweaksym$Subject.ID,dataweaksym$Cooperation.time)%*%diag(c(0,1,2, 3,4,5)))/rowSums(table(dataweaksym$Subject.ID,dataweaksym$Cooperation.time))- >weaksym

cbind(row.names(table(dataweaksym$Subject.ID,dataweaksym$Cooperation.time)),weaksy m,rep(0,60),rep(0,60))->weaksym2

rowSums(table(datastrongsym$Subject.ID,datastrongsym$Cooperation.time)%*%diag(c(0,1, 2,3,4,5)))/rowSums(table(datastrongsym$Subject.ID,datastrongsym$Cooperation.time))- >strongsym

cbind(row.names(table(datastrongsym$Subject.ID,datastrongsym$Cooperation.time)),strong sym,rep(1,60),rep(0,60))->strongsym2

rowSums(table(dataweakasym$Subject.ID,dataweakasym$Cooperation.time)%*%diag(c(0,1 ,2,3,4,5)))/rowSums(table(dataweakasym$Subject.ID,dataweakasym$Cooperation.time))- >weakasym

cbind(row.names(table(dataweakasym$Subject.ID,dataweakasym$Cooperation.time)),weak asym,rep(0,60),rep(1,60))->weakasym2

rowSums(table(datastrongasym$Subject.ID,datastrongasym$Cooperation.time)%*%diag(c(0 ,1,2,3,4,5)))/rowSums(table(datastrongasym$Subject.ID,datastrongasym$Cooperation.time)) ->strongasym

cbind(row.names(table(datastrongasym$Subject.ID,datastrongasym$Cooperation.time)),stro ngasym,rep(1,60),rep(1,60))->strongasym2

rbind(weaksym2,strongsym2,weakasym2,strongasym2)->data2

data.frame(data2)->data2

names(data2)<-c("Subject.ID","mean","Players.type","Game.type")

as.numeric(as.character(data2$mean))->data2$mean

global.model<- lmer(mean~Game.type*Players.type+(1|Subject.ID),data = data2,na.action = "na.fail",REML=F)

stdz.model <- standardize(global.model,standardize.y = FALSE) model.set <- dredge(stdz.model) top.models <- get.models(model.set, subset = delta<2) model.avg(top.models)->m1

#Model 2

read.csv(file="Data.csv",head=TRUE,sep=",")->data

data[data$Player.OptOut==0,]->data

data[data$Partner.OptOut==0,]->data

data[data$Player.Cooperated.More.than.Partner==1,]- >dataPlayer.Cooperated.More.than.Partner

global.model<- glmer(Player.Punished~Game.type*Players.type+(1|Subject.ID),family=binomial,data = dataPlayer.Cooperated.More.than.Partner,na.action = "na.fail",control=glmerControl(optimizer="bobyqa"))

stdz.model <- standardize(global.model,standardize.y = FALSE) model.set <- dredge(stdz.model)

top.models <- get.models(model.set, subset = delta<2) stdz.model->m2

#Model 3

read.csv(file="Data.csv",head=TRUE,sep=",")->data

data[data$Player.OptOut==0,]->data

data[data$Partner.OptOut==0,]->data

data[data$Player.OptOut.Prev==0,]->data

data[data$Partner.OptOut.Prev==0,]->data

data[complete.cases(data$Increased),]->data

data[complete.cases(data$Partner.Punished.Prev),]->data

data[data$Player.Cooperated.Less.than.Partner.in.Prev==1,]- >dataPlayer.Cooperated.Less.than.Partnerlast

global.model<- glmer(Increased~Partner.Punished.Prev*Game.type*Players.type+Partner.Punished.Prev+( 1|Subject.ID),family=binomial,control=glmerControl(optimizer="bobyqa"), data = dataPlayer.Cooperated.Less.than.Partnerlast,na.action = "na.fail")

stdz.model <- standardize(global.model,standardize.y = FALSE) model.set <- dredge(stdz.model) top.models <- get.models(model.set, subset = delta<2) model.avg(top.models)->m3

#Model 4

read.csv(file="Data.csv",head=TRUE,sep=",")->data

data[data$Player.OptOut==0,]->data

data[data$Partner.OptOut==0,]->data

data[data$Player.OptOut.Prev==0,]->data 13

data[data$Partner.OptOut.Prev==0,]->data

data[complete.cases(data$Partner.Punished.Prev),]->data

data[data$Player.Cooperated.Less.than.Partner.in.Prev==1,]->data

data[data$Player.Cooperated.Less.than.Partner==1,]- >dataPlayer.Cooperated.Less.than.PartnerlastPlayer.Cooperated.Less.than.Partnernow

global.model<- glmer(Player.Punished~Partner.Punished.Prev*Game.type*Players.type+(1|Subject.ID),famil y=binomial, data = dataPlayer.Cooperated.Less.than.PartnerlastPlayer.Cooperated.Less.than.Partnernow,na.a ction = "na.fail",control=glmerControl(optimizer="bobyqa"))

stdz.model <- standardize(global.model,standardize.y = FALSE) model.set <- dredge(stdz.model) top.models <- get.models(model.set, subset = delta<2) model.avg(top.models)->m4

confint(m4)

#Model 5

read.csv(file="Data.csv",head=TRUE,sep=",")->data

global.model<- glmer(Player.OptOut~Players.type*Game.type+(1|Subject.ID),family=binomial, data = data,na.action = "na.fail",control=glmerControl(optimizer="bobyqa"))

stdz.model <- standardize(global.model,standardize.y = FALSE) model.set <- dredge(stdz.model) top.models <- get.models(model.set, subset = delta<2) stdz.model->m4

#Model 6 read.csv(file="Data.csv",head=TRUE,sep=",")->data 14

data[data$Player.OptOut.Prev==0,]->data

data[data$Partner.OptOut.Prev==0,]->data

data[complete.cases(data$Partner.Punished.Prev),]->data

data[data$Player.Cooperated.Less.than.Partner.in.Prev==1,]->data

data[data$Partners.type==1&data$Players.type==0,]->data

global.model<- glmer(Player.OptOut~Partner.Punished.Prev+(1|Subject.ID),family=binomial, data = data,na.action = "na.fail",control=glmerControl(optimizer="bobyqa"))

stdz.model <- standardize(global.model,standardize.y = FALSE) model.set <- dredge(stdz.model) top.models <- get.models(model.set, subset = delta<2) stdz.model->m6
