# Supplementary material for: Power Asymmetries and Punishment in a Prisoner’s Dilemma with Variable Cooperative Investment
Source: PLoS One. 2016 May 18;11(5):e0155773. doi: 10.1371/journal.pone.0155773 (PMC4871419; doi:10.1371/journal.pone.0155773)
Supplement: S1 Fig — (DOC) [file pone.0155773.s006.doc]

**S1 Figure.**

**
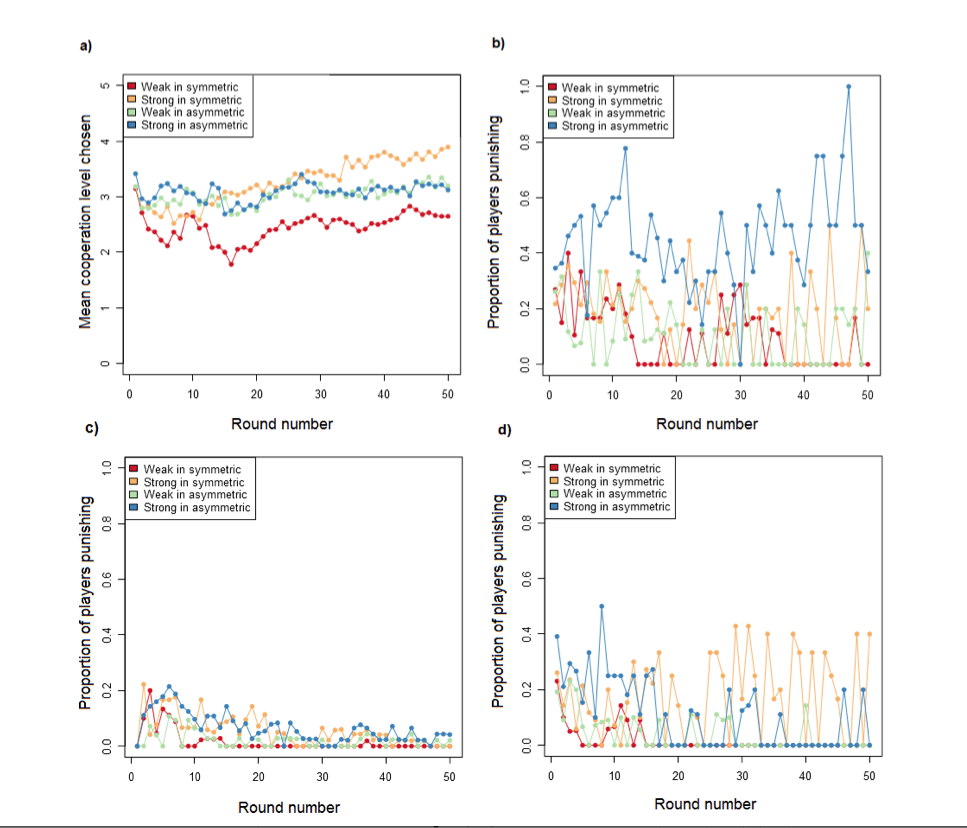
**

**S1 Figure.** Scatter plot showing a) the mean cooperation level chosen; and the mean proportion of players that chose b) justified punishment c) hypocritical punishment and d) antisocial punishment in each round according to whether they were weak or strong and whether they were in a symmetric or asymmetric game. Rounds where either player opted out were excluded.
